# Presumptive Tests for Xylazine—A Computer Vision Approach

**DOI:** 10.1002/ansa.70008

**Published:** 2025-04-01

**Authors:** Hui Yun Chang, Kristin Donnachie, Timothy J. D. McCabe, Henry Barrington, Felicity Carlysle‐Davies, Kristin Ceniccola‐Campos, Marc Reid

**Affiliations:** ^1^ Department of Pure and Applied Chemistry University of Strathclyde Glasgow UK

**Keywords:** cameras, computer vision, forensics, reaction monitoring, spot test

## Abstract

Abuse of xylazine is an immediate global public health concern. We report the distinct and measurable colour changes when xylazine is exposed to the Mandelin, Marquis and Mecke presumptive test reagents. The colour changes observed with xylazine are distinct from those of drugs that give colour changes from the same presumptive tests. To overcome the subjective limitations of determining spot test results by‐eye, we applied image and video analyses to quantify the distinctive features of presumptive tests with xylazine and thus differentiate it from other illicit substances tested under the same conditions, including morphine, fentanyl, heroin and methamphetamine. Herein, experimental protocols utilising *Kineticolor*, a computer vision software, were developed to qualitatively and quantitatively study presumptive tests for xylazine detection. To the best of our knowledge, these findings represent the first presumptive test strategy towards specific, quantifiable and potentially field‐ready detection of xylazine.

## 1 Introduction

### 1.1 Xylazine

**FIGURE 1:**
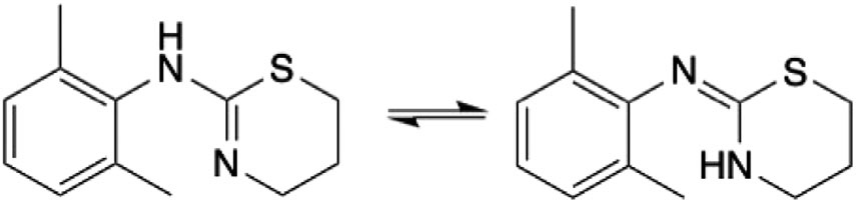
The chemical structure of xylazine in both its tautomeric forms; C_12_H_16_N_2_S.

Xylazine (Figure 1) is a non‐opioid tranquillizer developed for veterinary medicine. It is an alpha‐2 receptor agonist, acting as a muscle relaxant and used to sedate large animals [1, 2]. As a potent vasoconstrictor, xylazine can result in skin ulcers as a side effect of consumption, leading to the formation of necrotic tissue at the injection. Hence, xylazine is also known as the ‘zombie drug’ [3, 4]. Xylazine has not been approved for safe use in humans by any healthcare authorities including the UK's Medicines and Health Care Products Regulatory Agency (MHRA), the European Medicines Agency (EPA) and the United States Food and Drug Administration (FDA) [5, 6, 7]. However, people can be exposed to this drug either accidentally or intentionally, often in combination with other illicit drugs. Xylazine can be injected, snorted, inhaled or swallowed.

Xylazine was first detected in illicit drug markets in Puerto Rico in 2001 after several xylazine‐associated human deaths were reported by the local criminal hospital [8, 9, 10]. It was commonly detected in mixtures with various drugs, including cocaine, benzodiazepines, heroin and alcohol. Some mixtures are akin to the well‐known ‘speedball’ (mixture of cocaine and opioid) preparation [3]. The deliberate adulteration of various drugs with xylazine is done with the intent of enhancing the effect of depression on the central nervous system. Xylazine abuse can manifest in multiple symptoms including hypotension, bradycardia, hyperglycaemia or respiratory depression [1, 2].

According to Friedman et al. [3], the highest prevalence of xylazine‐associated deaths in the USA occurred in Philadelphia (26%), followed by Maryland (19%) and then Connecticut (10%) [3, 11]. In the UK, the first xylazine‐associated fatal case was reported by the National Programme on Substance Abuse Deaths in December 2022 [12]. As of August 2023, a collated post‐mortem toxicology report revealed that xylazine was detected in biological samples that caused 14 deaths in humans in the UK, demonstrating that xylazine has now deeply infiltrated the illicit drug market. Although xylazine has been reported to cause many deaths, it remains, at the time of writing, a non‐controlled substance under the Misuse of Drugs Act (1971). It is, however, categorised under the Psychoactive Substance Act (2016) due to its psychoactive effect [13, 14, 15]. Since xylazine has adulterant effects on a wide range of drugs, this has become an international public health concern [12].

### 1.2 Presumptive Testing

Colour‐changing presumptive tests (a.k.a. ‘spot’ tests) are one of the most common destructive methods used in a forensic laboratory to qualitatively detect the presence of a certain drug class in an unknown powder seized from the crime scene [16, 17]. Forensic scientists and field agents can rapidly identify the drug class of the illicit powder by observing the colour change after the seized sample has been exposed to a presumptive test reagent. Fundamentally, this technology is built on a chemical reaction occurring between the compounds in the test reagent and the active functional groups in the drug being tested. However, most mechanisms of the test reagents towards drugs are hypothesised and have not been definitively determined.

Due to their inability to guarantee sensitivity and specificity, spot tests cannot be used to confirm the identification and quantification of the drug. They are used as a guide for selecting the most appropriate confirmatory analysis such as gas chromatography (GC‐MS) or high‐performance liquid chromatography (HPLC) based on the detected drug classes. Although presumptive tests were classified by the Scientific Working Group for the Analysis of Seized Drugs (SWGDRUG) as the lowest discriminating power technique, they are still widely used by law enforcement and forensic laboratories due to their low cost and requiring only small (milligram) quantities of the drug [18, 19, 20].

Various well‐established test reagents are used in forensic laboratories, including Scott's, Marquis, Mandelin, Fast Blue B and Duquenois Levine reagents, plus many more [16]. Due to the perennial emergence of new psychoactive substances (NPS), these traditional test reagents are frequently modified so that they can be used to more selectively detect the specific drug class in the illicit powder. Mandelin, Marquis and Mecke reagents, primarily used to detect alkaloids such as amphetamines‐type stimulants (ATS), opium and synthetic cathinones, are used in the present study [21, 22, 23, 24]. The Mecke reagent is also primarily used to detect alkaloids such as some ATS, opioid drug classes and synthetic cathinones [22, 23, 24]. Selenious acid is the active compound triggering colour changes in the Mecke test [25]. Ammonium metavanadate is the main reacting compound in the Mandelin's reagent. The vanadium ions are known as ‘rainbow compounds’ due to the various colours at their different oxidation states [18]. Marquis reagent is mainly used to detect ATS and opium alkaloids where the combination of formaldehyde and concentrated sulphuric acid reacts with the drug molecules, forming a coloured compound. While these test results are typically determined by‐eye, camera technology has been explored as a ‘digital eye’ to bring more discriminating power to the accessible yet fallible spot test methodology.

### 1.3 Computer Vision in Analytical Chemistry

**FIGURE 2:**
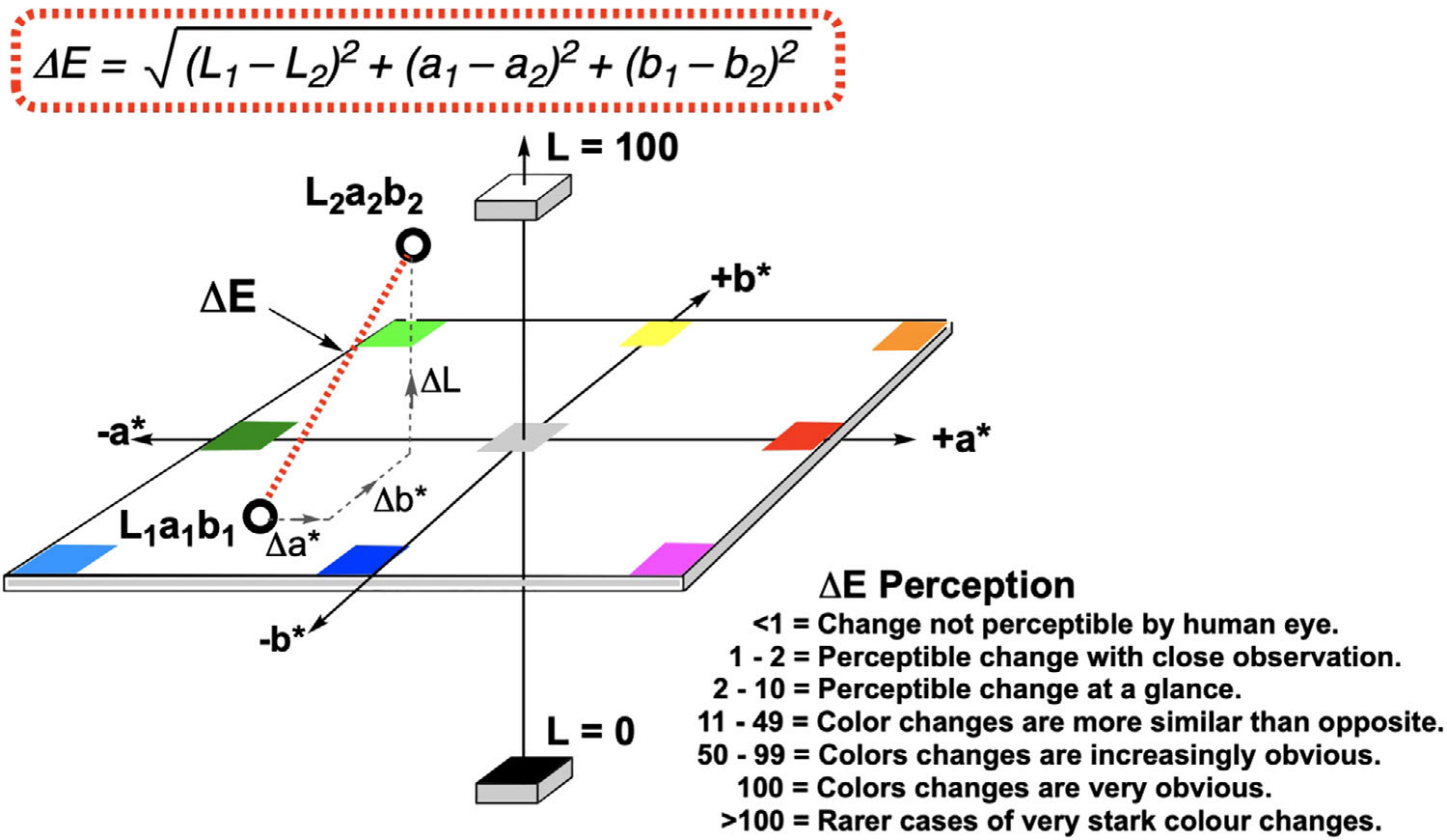
Simplified representation of the CIE–L*a*b* colour space. The L* axis represents lightness (higher values denote lighter colours); the a* axis represents the green‐to‐red colour spectrum (more positive values are more red); the b* axis represents the blue‐to‐yellow spectrum of colours (more positive values are more yellow). ∆*E*, in its simplest formulation, represents the Euclidean distance between two points in L*a*b* space and is thus a measure of contrast between any two colours.

In the chemistry context, a computer vision analysis system consists of an imaging device (typically a camera) and processing software that has been used to quantify colours from solutions recorded in videos and images [26]. The pixel data extracted from the sources enables colour quantification analysis by implementing multiple colour models such as RGB (red, green and blue), HSV (hue, saturation, value) and CIE–L*a*b* (lightness, green to red and blue to yellow) [26, 27, 28]. With such quantifiable colorimetric frameworks, computer vision has become a popular approach used for non‐contact monitoring and quantification of various chemical reactions [26, 28–30]. From our team's earlier forensic research [28], and in the current paper, the CIE–L*a*b* colour space represents the most intuitive quantification of colour and is designed to be perceptually uniform. Indeed, the ∆*E* metric that can be defined from a comparison of pairs of L*a*b* coordinates provides a valuable means of expressing colour‐agnostic contrast changes (Figure 2).

#### 1.3.1 Computer Vision in Forensic Science

The forensic science community is aware of the use of digital imaging to better quantify subjective colour tests that are traditionally analysed by‐eye or colour chart comparisons. The application of digital image analysis in drug detection was first introduced by Choodum et al. to analyse the products of presumptive colour tests for amphetamine, methamphetamine and opiates [31]. This has subsequently been supported by many other studies [31, 32, 33]. Digital image technology has evolved into an increasingly objective and specific analytical tool for presumptive testing. We [28], and others [34], have investigated computer vision technology to enable real‐time monitoring of the chemical reactions that underlie the presumptive test strategies. Similar technologies have been developed for other low‐cost sensing applications, including bubble counting [35] and atmospheric gas detection [36]. This digital and time‐resolved approach provides a more quantitative presumptive test based not only on the end result of the reaction but also on the kinetics of how it gets to the observed endpoint.

#### 1.3.2 Kineticolor

**FIGURE 3:**
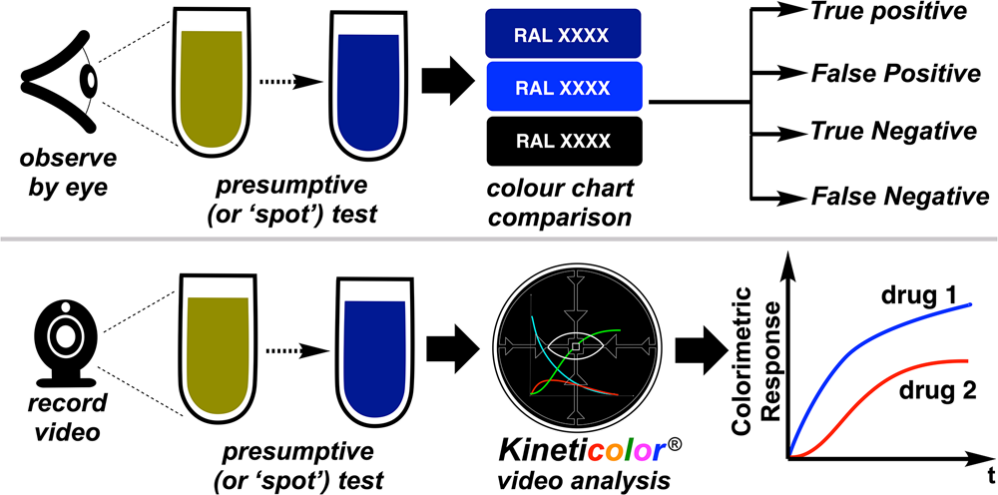
An overview of colour presumptive test with *Kineticolor*, adapted from [28].

For applications in and beyond forensic spot testing, our team has focused on the development of the computer vision software, *Kineticolor* [27, 28, 37, 38]. It enables non‐contact monitoring of chemical reactions by analysing video data, providing the rate of colour change and converting the values into other colour space models such as the HSV and the CIE–L*a*b* colour space. This software analyses both video footage and images of colorimetric reactions. ∆*E* (red dotted line and boxed equation in Figure 2) is the primary metric used in this study, though all other time‐resolved colour metrics are available in the supporting information. It is typically measured with a scale of 0 to approximately 100 where 0 shows no colour change versus the first video frame while 100 (or rarer examples >100) shows an obvious colour change. In our earlier forensics‐focused work, we studied the application of *Kineticolor* computer vision analysis for presumptive colour drug detection on amphetamine, barbiturates and benzodiazepines [28]. Results showed the potential of the computer vision analysis in enhancing the objectivity of forensic colour presumptive tests via recording the rate of colour change (∆*E* vs. time) as well as the final colour formed. The same time‐resolved method is applied in this study for real‐time monitoring of the colour change of multiple colour presumptive tests on xylazine (Figure 3).

## 2 Study Aims

At present, be it for biological or pure samples of the drug, presumptive testing for xylazine is limited to immunoassay test kits [39, 40]. This method is commonly used to detect the presence of drugs in biological samples such as hair, blood, urine and saliva. It is rarely used to detect drugs in their powder form„ which are the illicit drugs found in crime scenes or clandestine laboratories [41]. Lateral flow immunoassay test strips (XTS), as highlighted by Sisco et al. [40], provide a rapid means of drug checking, demonstrating high specificity and sensitivity for concentrations above 2 µg/mL. However, these immunoassays can produce false positives, notably with lidocaine. In contrast, our proposed computer vision approach (below) enables non‐contact analysis, offering a versatile and adaptable analytical solution for diverse sample types.

Xylazine has yet to be tested with various presumptive test reagents. To the best of the authors’ knowledge, there are no reported methods on presumptive or ‘spot’ tests for xylazine. Hence, this study aimed to investigate the potential application of Mandelin, Mecke and Marquis reagents in the presumptive testing for xylazine. In addition, we aimed to use image and video recordings to investigate the possible additional information that could be derived from *Kineticolor* and computer vision analysis of the same presumptive tests. This digital method quantifies colour changes in a time‐resolved manner, delivering not only end‐point analysis but also kinetic data, thus providing an added layer of specificity and reducing the risk of false positives.

## 3 Materials and Methods

### 3.1 Chemicals

Around 99% ammonium metavanadate was purchased from the Aldrich Chemical Company, Inc (USA). Concentrated sulphuric acid (approx. 95%) was purchased from the Fisher Chemical (UK). Around 97% selenious acid was produced from the Alfa Aeser (USA) and 37% formaldehyde was purchased from the Alfa Aeser (UK). Potassium hydroxide (pellets) and sodium hydroxide (pellets) were purchased from Fisher Chemical (Czech Republic). Petroleum ether of 40°C–60°C was purchased from Fisher Chemical (UK). Ethyl acetate was purchased from Sigma‐Aldrich (France). Two common contaminants (caffeine [42, 43] and sucrose [44]) found in illicit drugs were tested in this study. Caffeine was purchased from Sigma‐Aldrich (China) while sucrose (table sugar) was purchased from a local supermarket (Sainsbury's, Glasgow, UK). Drugs used in this research were xylazine, diazepam, amphetamine sulphate, morphine hydrochloride, MDMA, methamphetamine hydrochloride, lorazepam, oxazepam, cocaine hydrochloride, codeine, diamorphine hydrochloride and paracetamol (a.k.a acetaminophen). Xylazine (approx. 99%) was purchased from Sigma‐Aldrich (Switzerland). The commercial source of xylazine was used for all presumptive tests presented herein. The synthesis of xylazine is presented for completeness, as part of efforts to access the key thiourea intermediate (see below). All drugs tested, with the exception of xylazine, were obtained from the Centre of Forensic Science at the University of Strathclyde, UK. All work involving illicit substances was conducted in accordance with the University of Strathclyde's Home Office (UK) license requirements.

### 3.2 Reagent Preparation

#### 3.2.1 Presumptive Test Formulations

Mandelin and Mecke reagents were prepared according to a protocol reported by Carol et al. [45]. The Marquis reagent was prepared according to a protocol reported by Koole et al. [20].

#### 3.2.2 Hydroxide‐Containing Solutions

All hydroxide‐containing solutions were prepared with deionised water. Potassium hydroxide solution was prepared in a ratio of 1:12.5 while sodium hydroxide solution was prepared in a ratio of 1:8.9 (w/w). These solutions were used to trigger discolouration of Marquis Test samples to test for the presence of carbocation in the samples.

### 3.3 Synthesis of Xylazine

*NOTE—The following syntheses were carried out under controlled and licensed laboratory conditions. The synthesis of xylazine, or any other controlled substance, should not be attempted unless the appropriate government licence has been issued*.

**Intermediate thiourea A**. Acetone (25 mL) was dried over calcium chloride. Ammonium thiocyanate (5.30 g, 0.07 mol) was dried overnight in a vacuum oven set at 80°C. The dried ammonium thiocyanate was then dissolved in dry acetone in a 250 mL two‐necked round bottom flask which was fitted with a water condenser and a dropping funnel. The flask was then placed in an oil bath. Benzoyl chloride (6.7 mL) was added into the dropping funnel and added to the reaction mixture at a fast rate while the reaction was stirred at 600 RPM. The oil bath was then heated to above 100°C. Subsequently, 2,6 dimethylaniline (6.2 mL) was added to the dropping funnel and added to the reaction mixture in small portions, at a rate that made refluxing of the reaction mixture visible. Once the aniline was added in its entirety, the reaction was allowed to reflux for a further 5 min. The resulting reaction mixture was then cooled to approximately 10°C using a cold water ice bath. The resulting precipitate was then filtered off and washed with deionised water. In a separate round bottom flask, sodium hydroxide (7.5 g) was dissolved in deionised water (67.5 mL). The precipitate from the other flask was then added to the sodium hydroxide solution. The mixture was then boiled for 5 min to remove the benzoyl‐protecting group. The solution was then cooled to room temperature, and the resulting solid was filtered off and washed with water. The washed solid was then placed in the vacuum oven overnight to dry. This reaction produced a pale yellow free flowing solid (6.04 g, 0.03 mol, 66.6%). 1H NMR (500 MHz, DMSO‐d_6_, mixture of E and Z isomers): *δ* 9.15 (s, 0.6 H, isomer 1), 8.84 (s, 0.4 H, isomer 2), 7.50–7.29 (m, approx. 1.5 H, isomer 1), 7.12–7.05 (m, 3H, ArH for isomer 1 + isomer 2), 6.25 (bs, approx. 0.5 H, isomer 2), 2.17 (s, 6H, CH_3_ for isomer 1 + isomer 2) [46].

**Xylazine, from intermediate A**. N‐(2,6‐dimethylphenyl)thiourea (2.202 g) was added to a Schlenk tube along with 3‐amino‐1‐propanol (1.2 mL) and heated to above 125°C for 2 h and 45 min. The resulting mixture was removed from the heat and allowed to cool to room temperature before conc. HCl (2.6 mL) was added to the reaction. The reaction mixture was then refluxed for 30 min. The flask was then placed into an ice bath to cool. Deionised water (approx. 25 mL) was added to the mixture to precipitate out impurities, the resulting impurities were then filtered out with a further approx. 5 mL of water to wash the reaction vessel and filter cake. Subsequently, 32%–34% ammonia (10 mL) was added to the filtrate drop‐wise. The solution was left for 30 min to allow precipitation to occur. The resulting solid was then filtered and washed with water. The crude solid was recrystallised from hot ethanol to produce large white crystals of the xylazine‐free base (0.185 mg, 0.83 mmol, 6.87%). 1H NMR (500 MHz, DMSO‐d_6_):

7.32 (bs, 1H), 6.93–6.92 (d, 2H, 3J*_H H_* = 7.5 Hz), 6.77–6.74 (t, 1H, 3J*_H H_* = 7.5 Hz), 3.24 (apparent s, 2H), 2.91 (apparent s, 2H), 2.04 (s, 6H), 1.92 (pent., 2H, 3J*_H H_* = 6.0 Hz).

### 3.4 Presumptive Test Procedure

**FIGURE 4:**
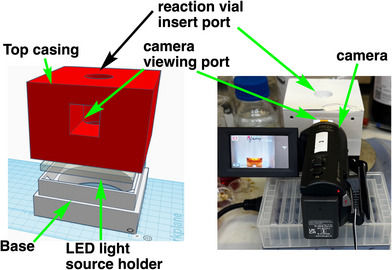
Left: 3D rendering of CAD drawing for 3D printed light box and reaction vial holder for quantitative and time‐resolved presumptive testing. Left: 3D rendering of the CAD design. Right: A photograph of the printed box in use.

A total of 10 drops of prepared Mandelin reagent were added into an empty 4 mL vial with a glass pipette which acted as a negative control. The glass vial was placed in a custom light box with a white background and a round Kodak LED light source on the base (Figure 4). A 6 × 3.5 mm cylindrical magnetic stir bar (Fisher brand) was used to thoroughly mix the sample and reagent at 300 RPM after the addition of the reagent. A Panasonic V180 camera was placed at a parallel angle to record the reaction from the side of the glass vial through a window in the lightbox. The setup is shown in Figure 4. Around 2 mg of one of the powdered samples listed above was added to the vial. Video recording was started before adding the reagent to capture the immediate colour change of the reagent once the reagent was added to the glass vial. The reaction was allowed to run for 5 min. The same procedures were followed when testing the Mecke and Marquis reagents with the other samples.

#### 3.4.1 Robotic Exploration of Xylazine Sample Concentrations

Using a 24‐well plastic well plate (4000 *µ*L wells), pairs of wells were charged with 1, 3 and 5 mg of pure xylazine. Using an OpenTrons (OT‐2) liquid handling robot, a protocol was created to dispense the Marquis, Mandelin and Mecke reagent formulations into the wells. Reservoirs of each reagent were transferred to beakers held in a custom 3D‐printed holder mounted onto a deck position in the robot. All liquid transfers were carried out using the OT‐2 P1000 Gen2 single channel pipette and OT‐2 1000 *µ*L filter pipette tips, stored in a 96‐tip rack on the deck of the robot. To effectively mix the solid samples with the reagents charged into each well, the well plate was held using an OpenTrons 96 flat bottom heater‐shaker adaptor inserted into the OpenTrons Gen1 Heater‐Shaker module. The resulting assay tested all four conditions (including wells containing reagent only) being tested in duplicate. A top‐down photograph of the well plate was analysed to generate ∆*E* values for each test well relative to its respective blank (reagent‐only) well, according to the procedure in Section 3.6.2.

### 3.5 Imaging Methods

A Panasonic V180 camera was used and was set to automatic mode with auto white balance, 1/50 shutter and 1080p resolution for video recording. The camera was placed approximately 0.5 cm away from the observatory window of the 3D‐printed light box and it was magnified to 6× to focus on the vial. This allowed the reactions in the vial to be observed clearly and to easily select a region of interest (ROI) for analysis in the *Kineticolor* software (version 0.3.2).

### 3.6 Kineticolor Analysis

#### 3.6.1 Video Analysis

**FIGURE 5:**
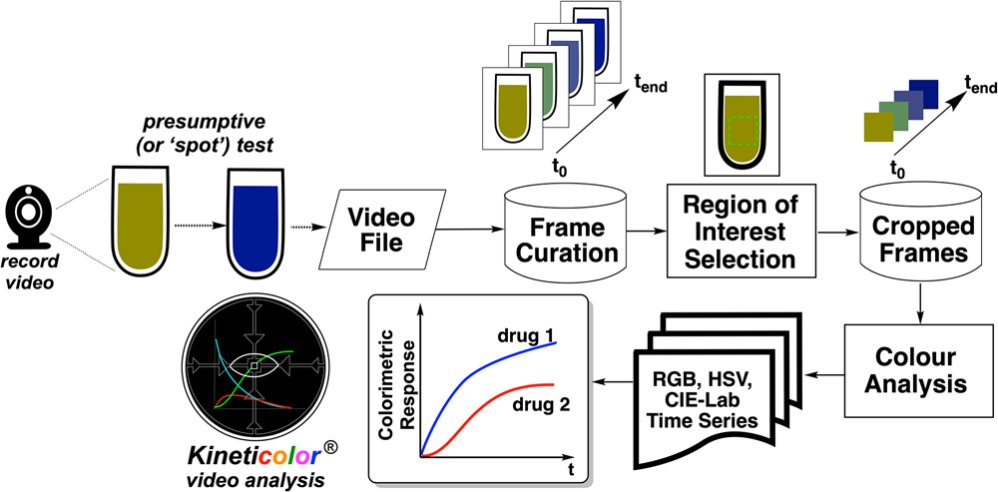
Analytical workflow of time‐resolved quantitative presumptive testing with *Kineticolor*.

The recorded videos of every sample were loaded onto the *Kineticolor* software for analysis. Frame curation was followed by setting the periodicity of frames to be ignored in the analysis (Figure 5). Then, a region of interest (ROI) in the video was selected by the user. The software then analysed the ROI and ignored the remainder of the frame outside of the ROI. The analysed frames were presented as plots of various colour parameters versus time. Additional details on the operation and underlying principles of this computer vision approach can be found in the reference [28]. All machine‐readable outputs from *Kineticolor* are available in a zipped folder on the figshare repository at: https://doi.org/10.6084/m9.figshare.26564395.v2.

#### 3.6.2 Image Analysis

Where single images were recorded, *Kineticolor* software was used to extract RGB, HSV and CIE–L*a*b* values from the selected region(s) of interest in the image. The raw quantified outputs of presumptive tests were then made relative for comparison by calculating ∆*E* with respect to vials containing the same volume of reagent only.

## 4 Results and Discussion

### 4.1 Three Spot Tests Are Better Than One

Our study began by testing a range of white powdered samples with each prepared reagent before testing with xylazine itself. Where relevant, all experimental results matched the reported outcomes, except for methamphetamine with the Mecke reagent for which, in our hands, a colourless to grey‐green result was recorded, versus the negative (no reaction) result reported in the literature (Table 1) [47]. We suspect that the subtlety of the colour change observed could be interpreted as there is no reaction, hence the subjective by‐eye disagreement between our result and that previously reported.

**TABLE 1:** Powdered samples tested with Mandelin, Mecke and Marquis reagents; (+) = detectable colour change by‐eye, (−) = no detectable colour change by‐eye.

Drug Sample	Mandelin	Marquis	Mecke
Xylazine	(+)	(+)	(+)
Caffeine	(−)	(−)	(−)
Methamphetamine hydrochloride	(+)	(+)	(+)
MDMA	(+)	(+)	(+)
Morphine hydrochloride	(+)	(+)	(+)
Amphetamine sulphate	(+)	(+)	(−)
Diazepam	(−)	(−)	(−)
Sucrose	(−)	(+)	(+)
Lorazepam	(+)	(+)	(+)
Oxazepam	(+)	(+)	(+)
Cocaine hydrochloride	(−)	(−)	(−)
Codeine	(+)	(+)	(+)
Diamorphine hydrochloride	(+)	(+)	(+)
Paracetamol (acetaminophen)	(+)	(+)	(+)

**FIGURE 6:**
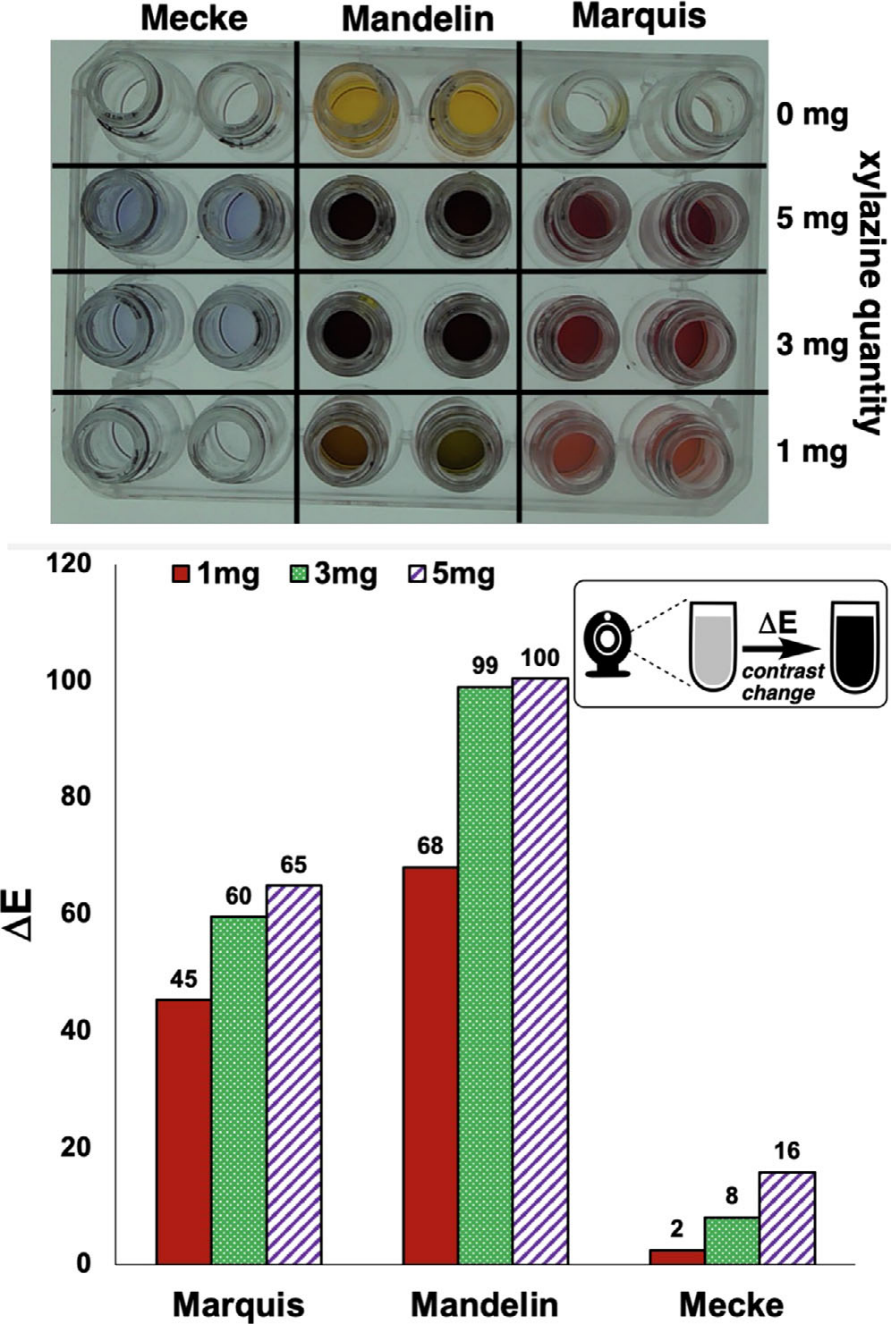
Presumptive test assay using ∆*E* to determine the sensitivity of the Marquis, Mandelin and Mecke tests to various mass quantities of xylazine.

With a focus on xylazine specifically, we tested the reproducibility and sensitivity of each spot test by means of a high throughput liquid handling robot. From a single photograph of a well plate, taken after the reactions had been run, we calculated the ∆*E* contrast change, relative to the blank reagent‐only wells (Figure 6). From these experiments, the Mandelin and Marquis reagents showed high contrast colour changes for all quantities of xylazine tested. Conversely, the Mecke reagent showed far more subtle colour change responses, depending on the quantity of xylazine present.

Combinations of spot tests can improve the power of the method towards identifying any one drug sample, particularly when a single colorimetric assay lacks the specificity or sensitivity required to quickly characterise a drug from an unknown powder [48, 49]. Table 2 presents the combined colorimetric results of the drugs tested in the three‐component presumptive test assay. Each drug exhibited a distinct visual colour profile in each test except amphetamine and methamphetamine, which produced identical by‐eye results in the Marquis and Mandelin tests. Xylazine displayed a distinctive set of colours in the three tests compared to the other drugs listed in the table. It was suspected that the characteristic combination of colour changes to each test with xylazine was due to the presence and reaction of the isothiourea functional group, present only in xylazine and not featured in any of the other drugs tested. No other drugs that we tested showed the same combination of colour changes in these three colour presumptive tests as xylazine. Therefore, the combination of the Marquis, Mandelin and Mecke test results could serve as a characteristic marker for xylazine, enabling authorities or analysts to differentiate it from other drugs of abuse.

**TABLE 2:** Colorimetric results of various drugs in Marquis, Mandelin and Mecke tests.

Drug	Marquis	Mandelin	Mecke
Xylazine	Red	Yellow‐brown	Blue
Diazepam	No change	No change	No change
Amphetamine	Brown	Green	No change
Methamphetamine	Brown	Green	Grey‐green^a^
MDMA	Brown	Brown	Blue‐green
Morphine	Purple	Brown	Green
Lorazepam	Yellow	Yellow‐brown	Yellow
Oxazepam	Orange	Yellow‐green	Light yellow
Cocaine hydrochloride	No change	No change	No change
Codeine	Black	Black	Brown‐black
Diamorphine hydrochloride	Black	Green	Brown‐black
Paracetamol (acetaminophen)	Brown‐black	Brown‐black	Yellow‐brown

^a^ Could be interpreted, by‐eye, as no colour change.

### 4.2 Marquis Test

**FIGURE 7:**
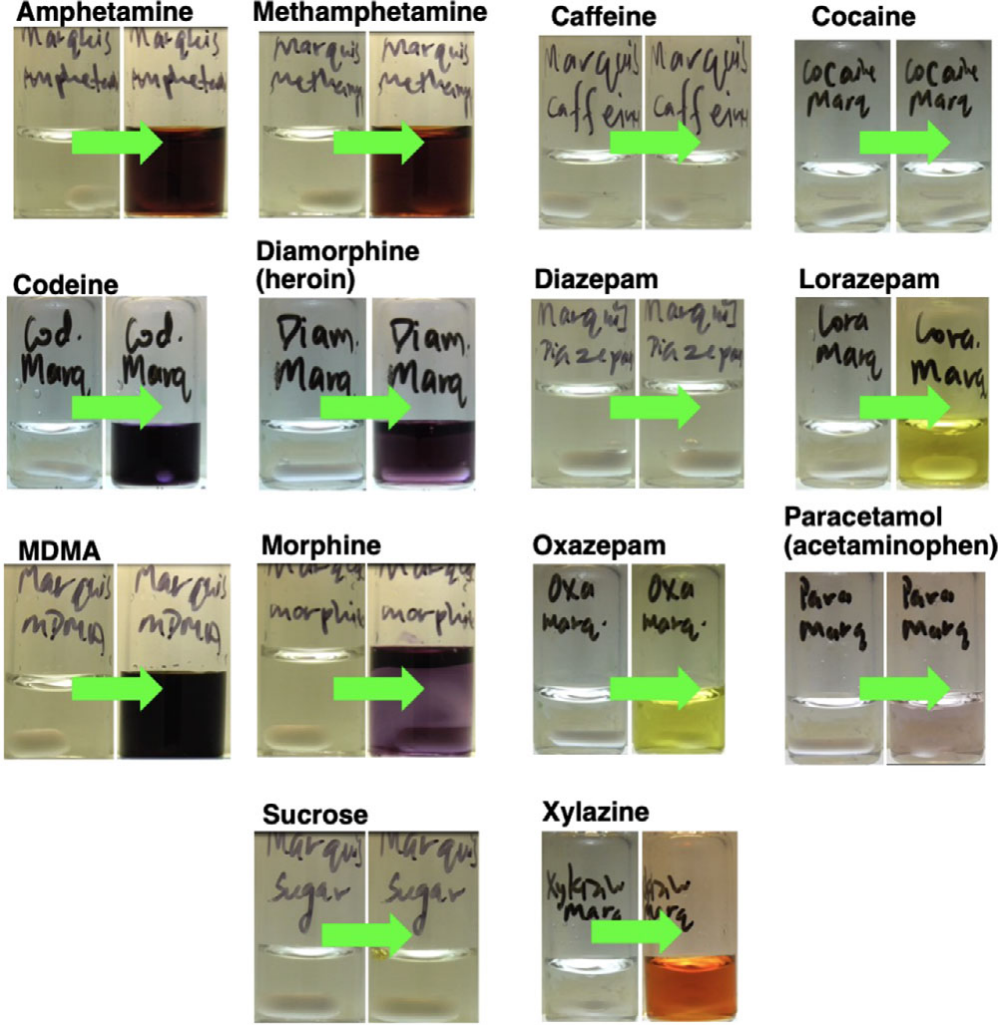
Colorimetric reactions on various powdered drug samples with the Marquis reagent.

Table 3 and Figure 7 show the colour changes of each white powdered sample tested with the Marquis reagent. It was noted that xylazine produced a distinct visible colour change (colourless to red, RAL 3000) compared to the other samples.

**TABLE 3:** Marquis test results observed visually were compared to the RAL colour chart.

Sample	Colour change	RAL (start)	RAL (end)
Xylazine	Colourless −→ Vermillion	9003	2002
Amph.H_2_SO_4_	Colourless −→ Black red	9003	3007
Methamph.HCl	Colourless −→ Black red	9003	3007
MDMA	Colourless −→ Grey brown	9003	8019
Morphine.HCl	Colourless −→ Claret violet	9003	4004
Sucrose	Colourless −→ Broom yellow	9003	1032
Caffeine	None	9003	9003
Diazepam	None	9003	9003
Lorazepam	Colourless −→ Curry yellow	9003	1027
Oxazepam	Colourless −→ Lemon yellow	9003	1012
Cocaine.HCl	None	9003	9010
Codeine	Colourless −→ Jet black	9003	9005
Diamorphine.HCl	Colourless −→ Black brown	9003	8022
Paracetamol	Colourless −→ Pearl beige	9003	1035

**FIGURE 8:**
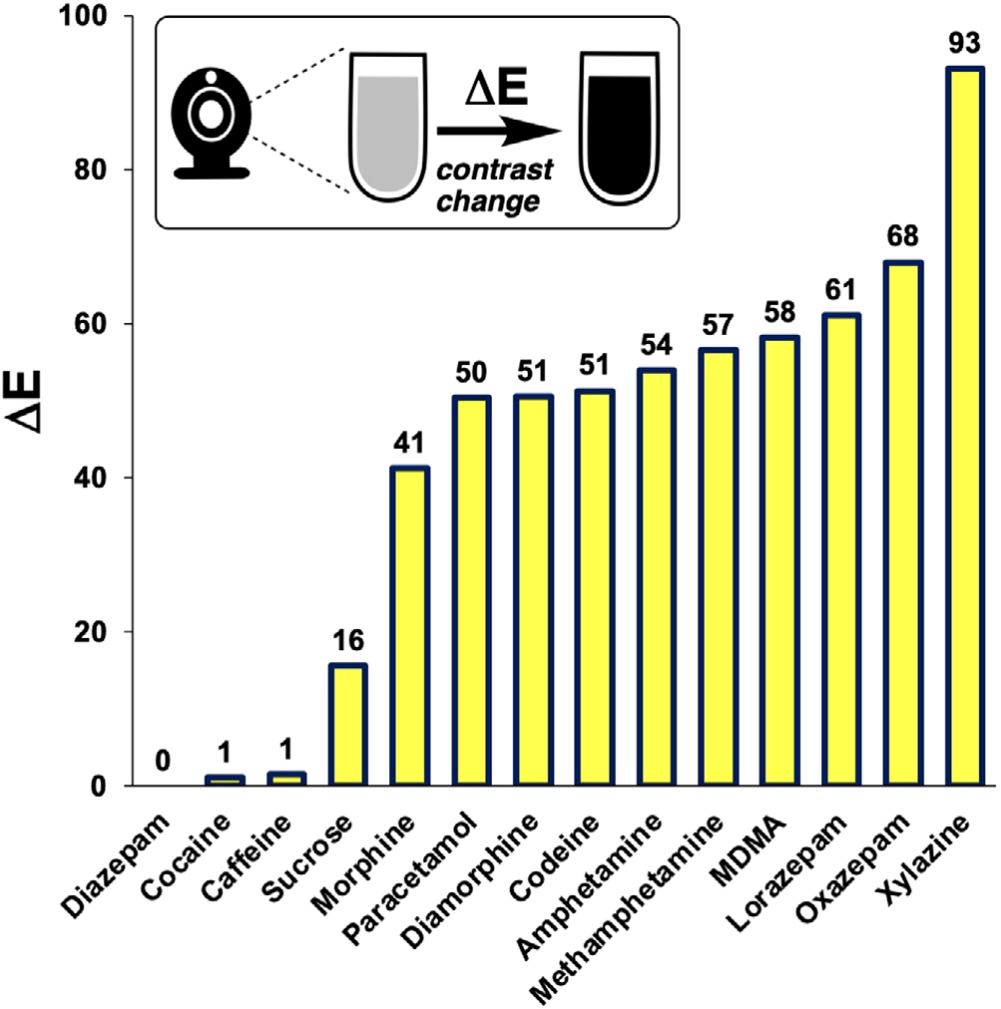
∆*E* of tested samples in Marquis test, relative to the starting colour before the reagent was exposed to the sample. Xylazine had the highest ∆*E* value indicated that the colour changes were obvious by‐eye. Diazepam, cocaine and caffeine resulted in the lowest ∆*E* values, indicating no colour change was observed.

The subjective visual observations from the Marquis test results were quantified by the numerical expression of colour analysed from still images, using *Kineticolor* (Figure 8). The numerical expressions of the colour at 0 s for all tested samples were similar in their values, representing the colourless blank reagent. The noticeable differences between the RGB values over time indicated an obvious colour change, as supported by the ∆*E* value. As the ∆*E* value increased, the colour change of the reagent can be observed more easily. Caffeine and diazepam had the lowest ∆*E* values at 1 and 0, respectively, consistent with there being no colour changes that could be observed by‐eye. In contrast, xylazine had the highest ∆*E* value at 93, indicating that the colour change was obvious and could be easily observed visually, which matched the result from visual observation shown in Table 3 and Figure 7. Figure 8 shows the range of ∆*E* values measured for samples tested with the Marquis reagent.

**FIGURE 9:**
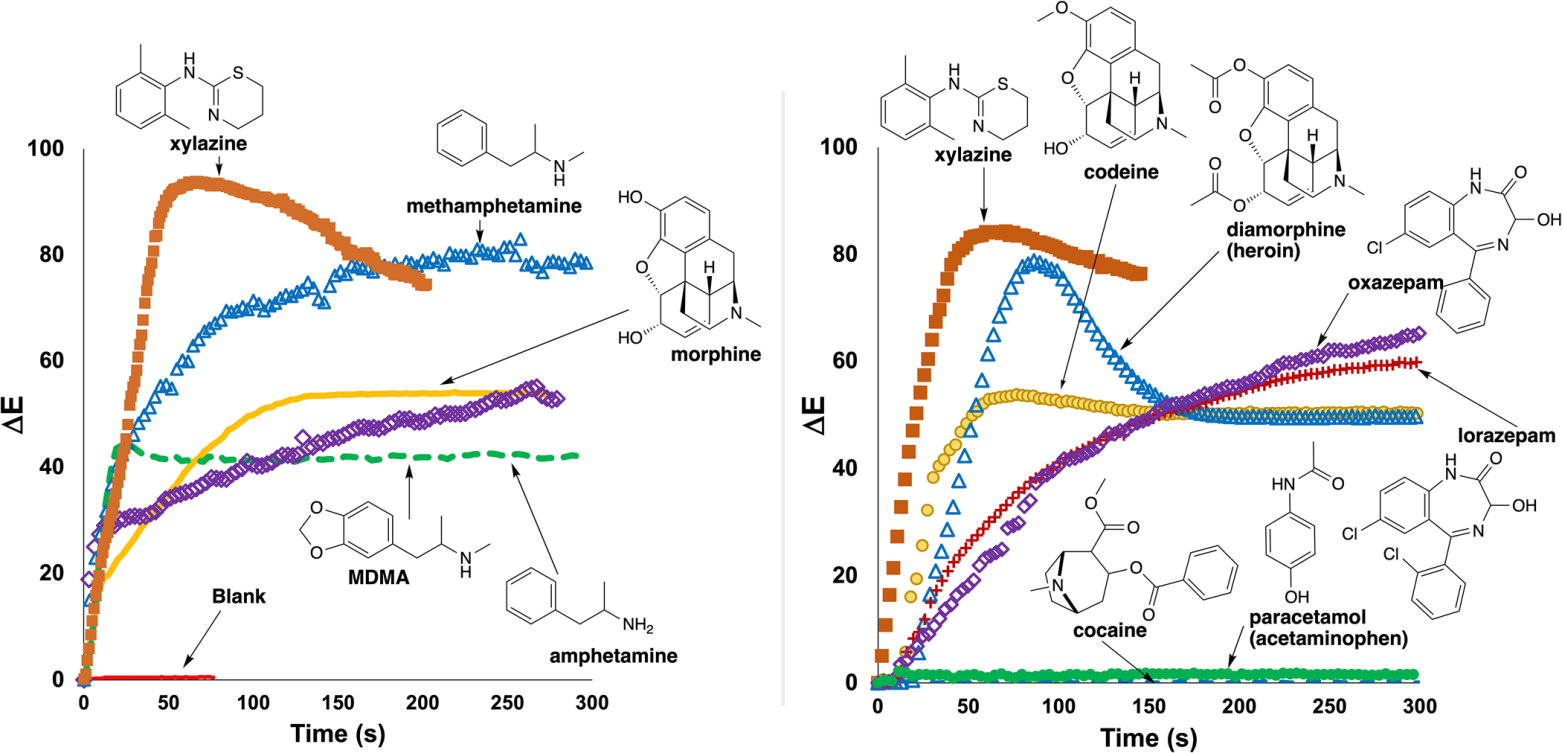
Comparison of the ∆*E* profiles of powdered samples from analysed video footage of Marquis tests. All drugs contained either a primary, secondary or tertiary amine functional group. Xylazine demonstrated a distinct ∆*E* profile compared to the other test samples.

The proof‐of‐concept exercise was expanded by using a video recording of each spot test to quantify the rate of colour change for powdered samples listed in Table 3 using the *Kineticolor* software. Xylazine yielded a distinctive ∆*E* profile over time compared to other drug samples, as shown in Figure 9. The colorimetric reactions for amphetamine, methamphetamine, MDMA and morphine resulted in observable colour changes by both visual and *Kineticolor* analysis. All samples contained either primary, secondary or tertiary amine. Diazepam and caffeine, in contrast, produced negative test results. Although sucrose is not an alkaloid, it is used as a cutting agent and is known to react with the Marquis reagent, causing false positive results [25].

**FIGURE 10:**
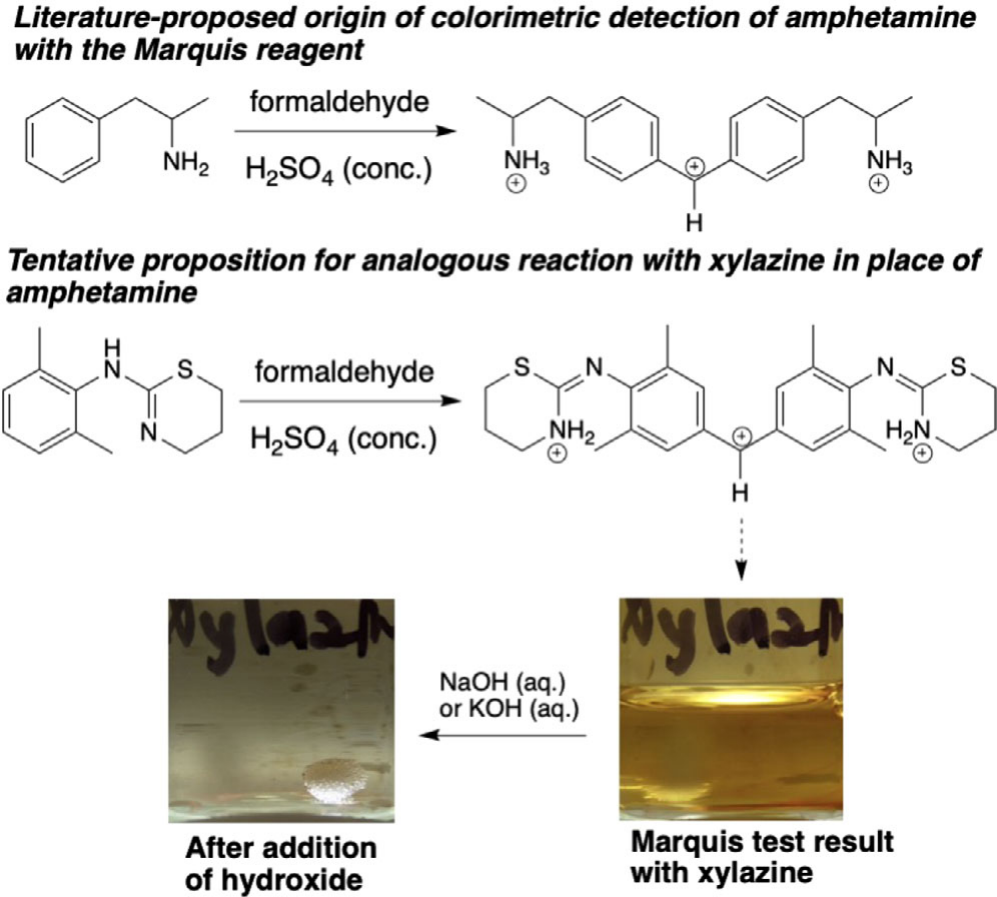
Top: Proposed mechanism of the root cause colour change in the Marquis test for amphetamine. A related benzylic carbocation may form when xylazine is exposed to the Marquis reagent. Bottom: Exposing the solution resulting from the Marquis test with xylazine to aqueous hydroxide results in the solution becoming colourless, qualitatively consistent with carbocation formation resulting from xylazine dimerisation during the Marquis test.

Previous research indicated that the combination of formaldehyde and concentrated sulphuric acid that constitutes that the Marquis reagent facilitates drug dimerization through the formation of a carbocation which is responsible for the observed colour change in positive Marquis tests [50]. This theory was applied in other research, which discovered that the colour formation by amphetamine in the Marquis test was due to the production of an orange carbocation ion from two amphetamine molecules and one formaldehyde as shown at the top of Figure 10 [16, 50]. Kovar and Laudszun reported the same mechanism in morphine detection. These suggest that the red product observed with xylazine may result from a dimer xylazine molecule responsible for colour formation. An extended Marquis test was conducted in this research, wherein sodium hydroxide or potassium hydroxide was added into the ‘red’ Marquis test solution to observe for any colour changes. The experiment demonstrated that the colour changed from red to colourless, consistent with a carbocation in the ‘red product’ (Figure 10, bottom). Further research is required to fully elucidate the likely mechanism(s) operating to give the positive Marquis test for xylazine.

### 4.3 Mandelin Test

**FIGURE 11:**
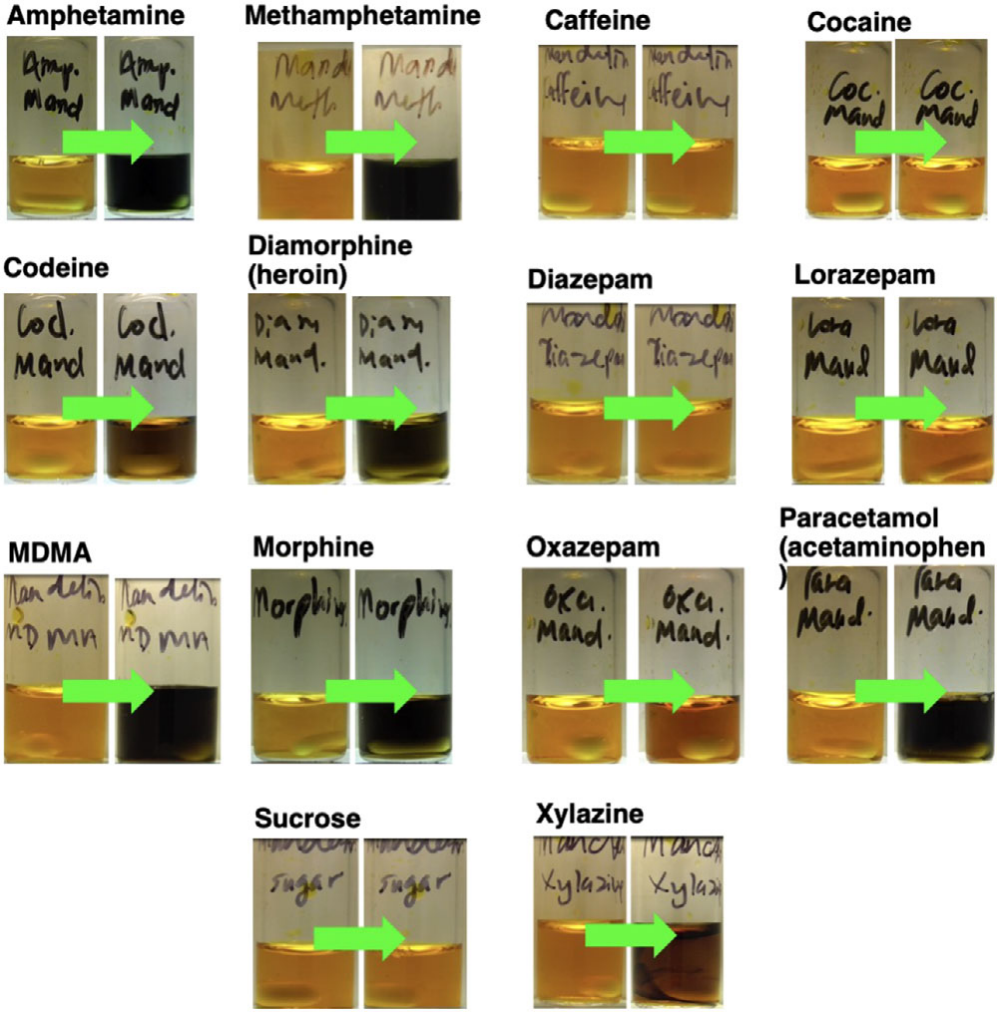
Colorimetric reactions of various powdered drug samples with the Mandelin reagent.

The same white powdered samples were tested with the Mandelin reagent. Four out of seven tested samples were known to show a positive colour change in the Mandelin reagent. The variety of colour changes of the reagent towards different samples can be observed in Figure 11 and Table 4.

**TABLE 4:** Mandelin test results of various powdered samples that were visually observed in Mandelin reagent were compared to the colour chart.

Sample	Colour change	RAL (start)	RAL (end)
Amphetamine	Yellow orange −→ Jet black	2000	9005
Methamphetamine	Yellow orange −→ Jet black	2000	9005
MDMA	Yellow orange −→ Jet black	2000	9005
Morphine	Yellow orange −→ Jet black	2000	9005
Sucrose	None	2000	2000
Xylazine	Yellow orange −→ Wine red	2000	3005
Caffeine	None	2000	2000
Diazepam	None	2000	2000
Lorazepam	None	2000	2000
Oxazepam	Yellow orange −→ Pearl orange	2000	2013
Cocaine.HCl	None	2000	2000
Codeine	Yellow orange −→ Black red	2000	3007
Diamorphine.HCl	Yellow orange −→ Black brown	2000	8022
Paracetamol	Yellow orange −→ Jet black	2000	9005

**FIGURE 12:**
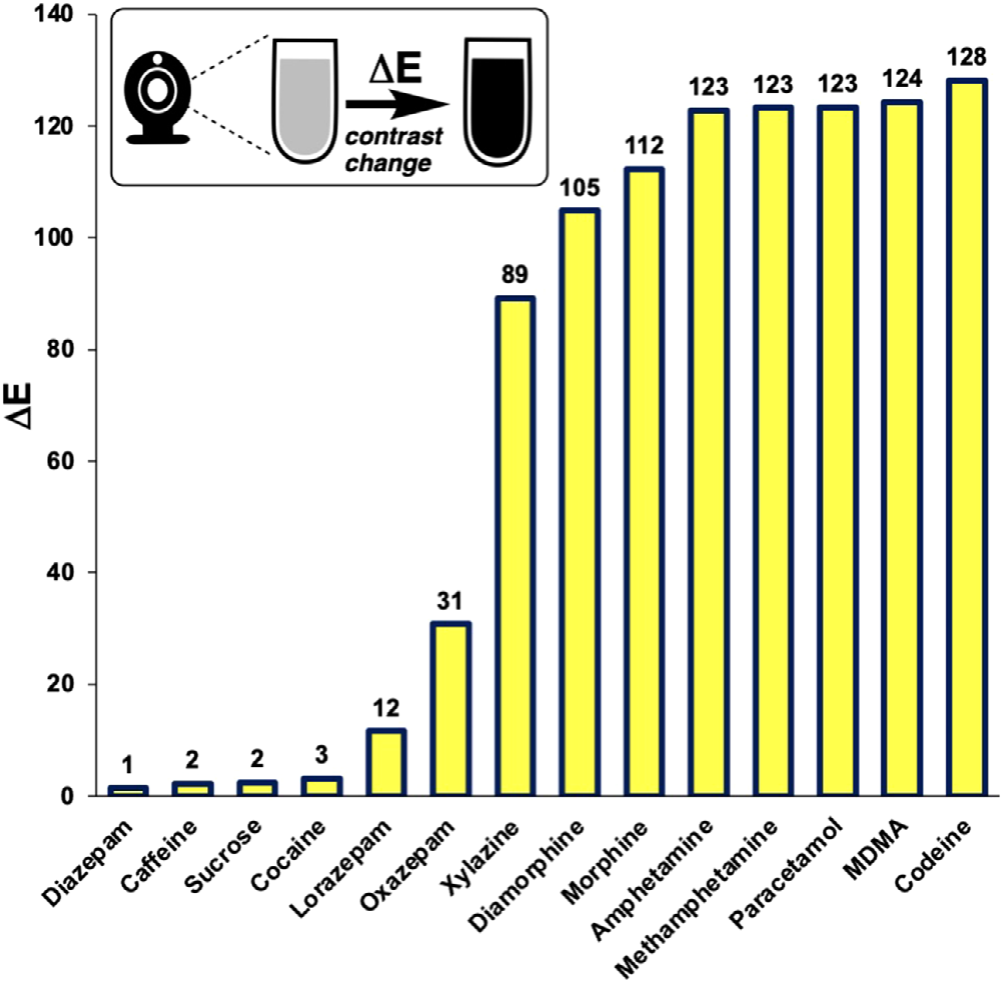
∆*E* of tested samples in Mandelin test, relative to the starting colour before the reagent was exposed to the sample.

These visually observed colour changes were supported by the numerical expression of the contrast change, ∆*E*, generated in *Kineticolor* (Figure 12). MDMA had the largest ∆*E* value of 124, indicating the most dramatic colour change that can be observed among the tested samples. In contrast, sucrose, caffeine and diazepam had the lowest ∆*E* values, indicating no observable colour change. The results also highlighted that xylazine had a large ∆*E* value of 89, supporting that it had an obvious colour change in the Mandelin test.

**FIGURE 13:**
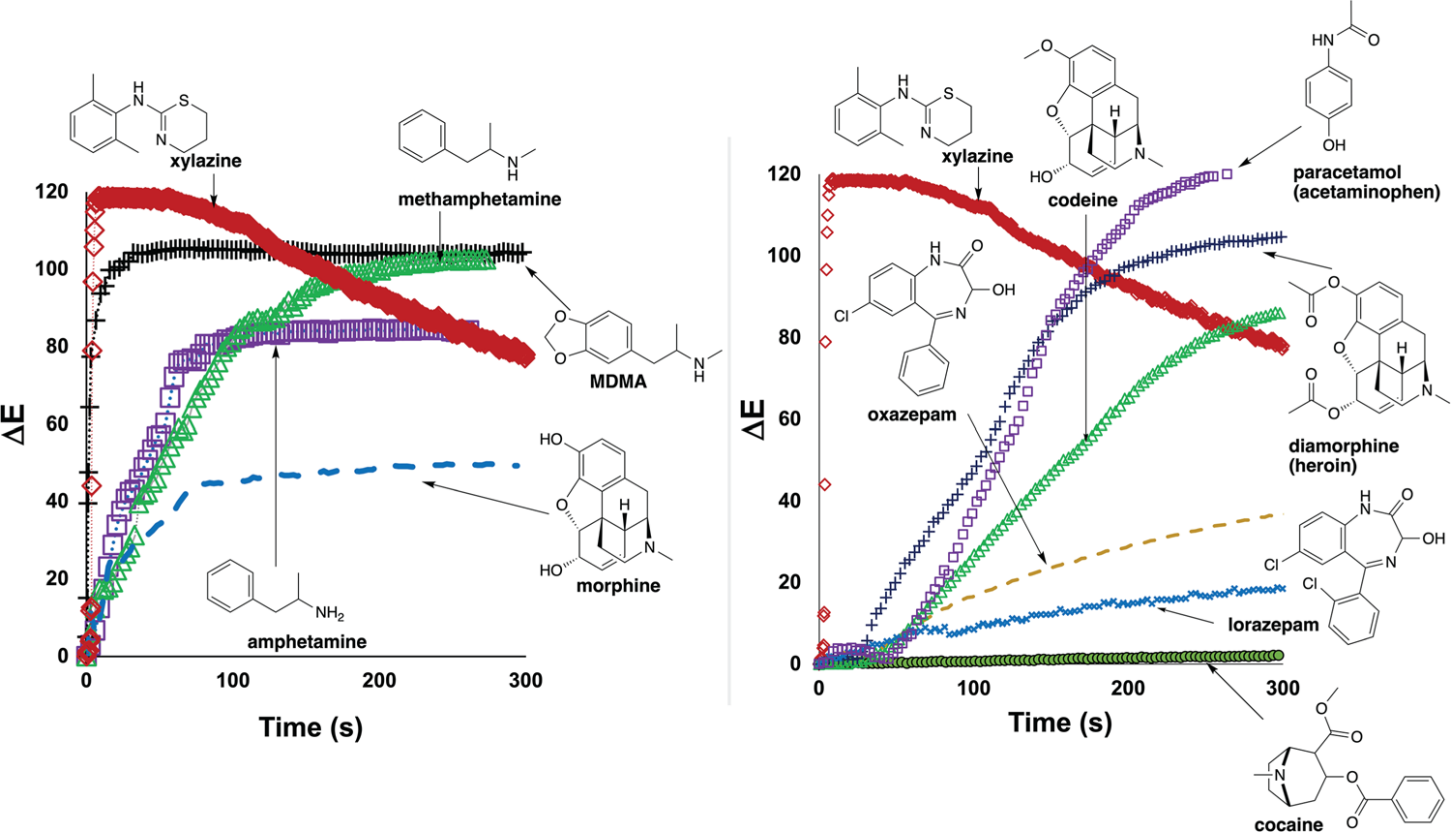
Comparison of the ∆*E* profiles for various powdered drug samples with Mandelin reagent. Xylazine demonstrated a distinctive ∆*E* profile compared to the other tested samples.

Like the Marquis reagent, the reactions of the tested samples were video recorded and analysed by *Kineticolor*, producing five distinctive ∆*E* profiles shown in Figure 13. MDMA and xylazine demonstrated the highest rates of colour change, followed by amphetamine, methamphetamine and morphine, each with progressively slower rates of colour change. This colour transition from purple to orange‐red and finally to yellow was demonstrated as the ∆*E* profile shown in Figure 13, with a gradual decrease in ∆*E* value over time. Nevertheless, xylazine exhibited a distinctive yellow solution formation and ∆*E* profile which could be used to differentiate it from other white powdered samples with Mandelin reagent.

### 4.4 Mecke Test

**FIGURE 14:**
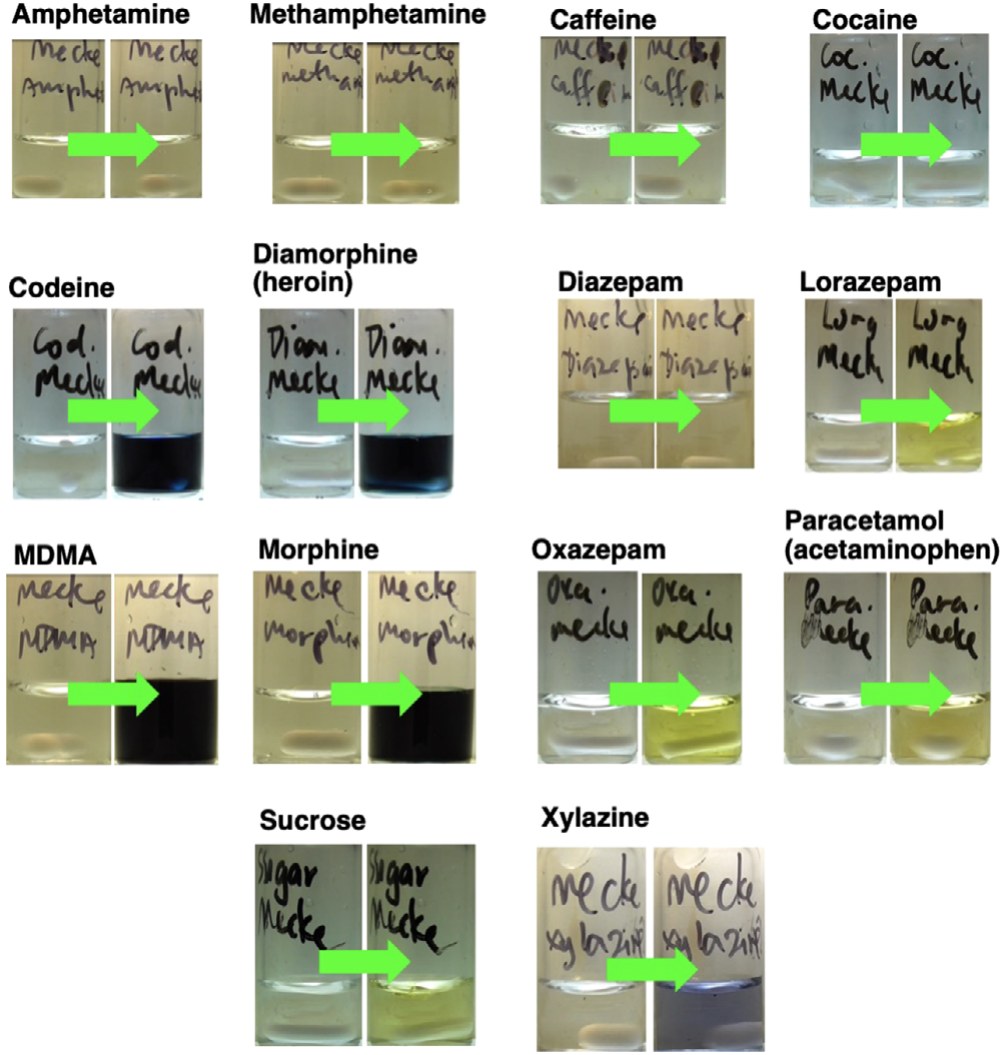
Colorimetric reactions of various powdered drugs with the Mecke reagent.

The last reagent used in this study was the Mecke reagent. Like the Mandelin reagent, the Mecke reagent showed positive colour changes for four of the seven tested white samples. The visual observation of the colour changes matched the test results from the published literature (Figure 14, Table 5), except for methamphetamine [25, 50, 51]. In our hands, methamphetamine had a colour change from colourless to pale green whereas this has previously been reported as producing no colour change [25].

**TABLE 5:** Mecke by‐eye results visually compared to the RAL colour chart.

Sample	Colour Change	RAL (start)	RAL (end)
Amphetamine	None	9003	9003
Methamphetamine	Colourless −→ Olive yellow	9003	6039
MDMA	Colourless −→ Blue‐green	9003	5020
Morphine	Colourless −→ Jet black	9003	9005
Sucrose	Colourless −→ Yellow green	9003	6018
Xylazine	Colourless −→ Grey blue	9003	5008
Caffeine	None	9003	9003
Diazepam	None	9003	9003
Lorazepam	Colourless −→ Curry yellow	9003	1027
Oxazepam	Colourless −→ Lemon yellow	9003	1012
Cocaine.HCl	None	9003	9003
Codeine	Colourless −→ Sapphire blue	9003	5003
Diamorphine.HCl	Colourless −→ Sapphire blue	9003	5003
Paracetamol	Colourless −→ Curry yellow	9003	1027

**FIGURE 15:**
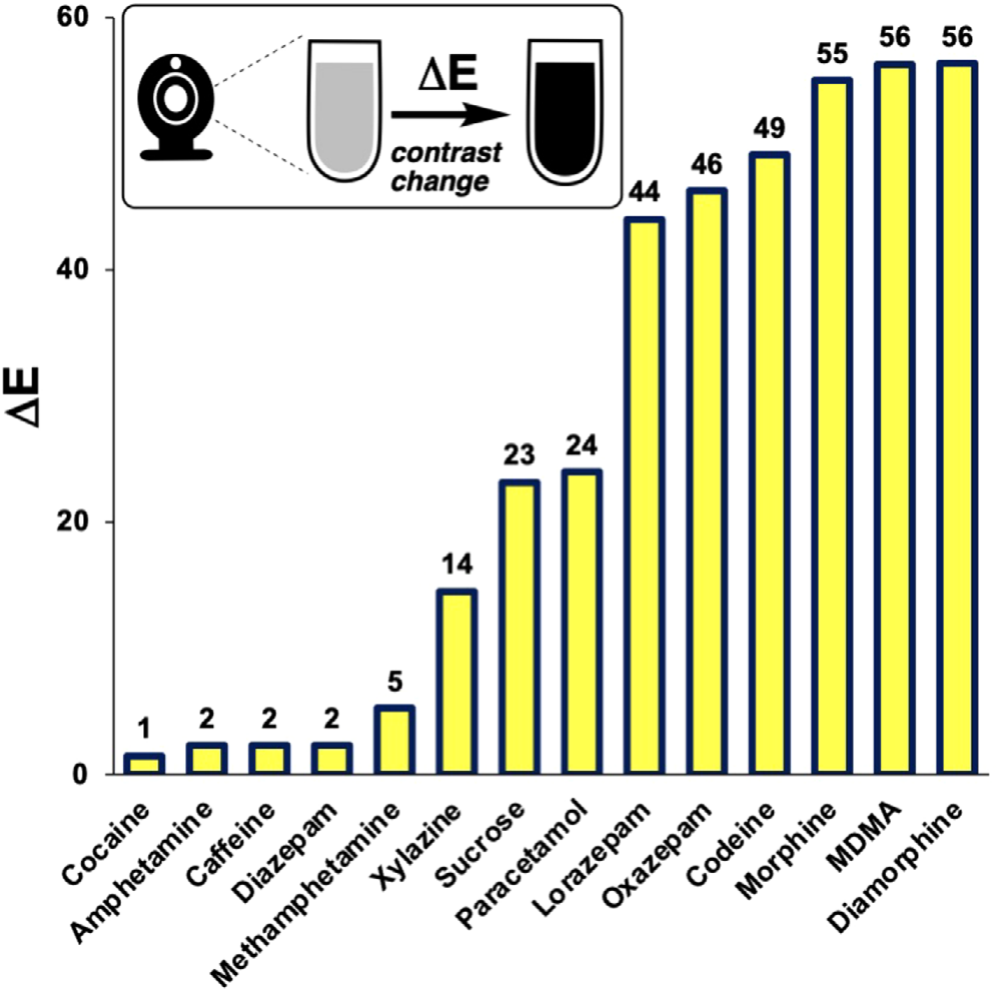
∆*E* versus tested samples for Mecke test. Morphine, diamorphine (heroin) and MDMA had the highest ∆*E* values, indicating that the colour changes were obvious by‐eye. Xylazine had a ∆*E* value about 25% that of the aforementioned drugs, indicating a more subtle colour change.

The colour change by methamphetamine in this study was not as pronounced as compared to morphine and MDMA when observed visually. Methamphetamine had a similar numerical expression of colour between 0 and 5 min and a small ∆*E* value of 5, shown in Figure 15. An obvious positive result should exhibit a distinctive increase or decrease in values and a large ∆*E* value such as those for morphine, MDMA and sucrose, both greater than 20. Amphetamine, caffeine and diazepam, expected to have negative test results had small ∆*E* values (less than 10), indicating no colour change. Xylazine had a moderate ∆*E* value of 14, indicating a perceptible but more subtle colour change than for sucrose, MDMA and morphine. The difference between the ∆*E* values is demonstrated in Figure 15. These results corresponded to the colour observed visually in Figure 14, Table 5.

**FIGURE 16:**
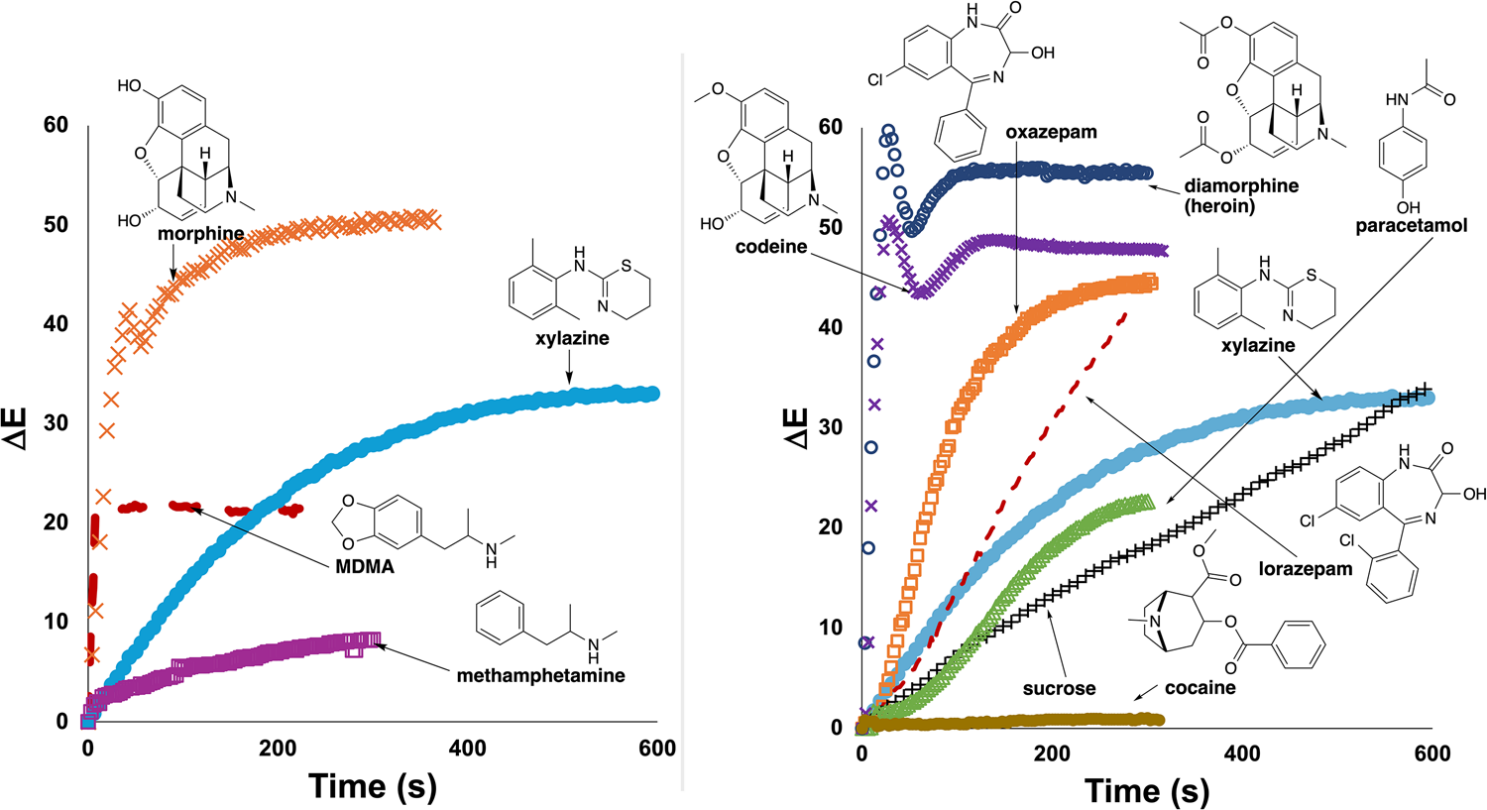
Comparison of the ∆*E* profiles for various powdered drug samples with Mecke reagent. Xylazine demonstrated a distinctive ∆*E* profile compared to the other tested samples.

When the colorimetric reactions were analysed by *Kineticolor*, distinctive ∆*E* profiles were obtained for all drug samples tested (Figure 16). For example, MDMA evidenced one of the highest rates of colour change, followed by morphine, xylazine and sucrose. Conversely, methamphetamine had a low rate of colour change, detectable despite methamphetamine being reported to have no colour change in the Mecke reagent [45, 47, 51]. This highlighted the capability of computer vision in capturing a subtle colour change in a chemical reaction which could be useful in detecting such colour change and can avoid any false negative result by visual observation in future tests. On the other hand, a distinctive blue colour (RAL 5023) was visually observed in xylazine's reaction at three minutes, giving a distinct ∆*E* profile compared to the other powdered samples (Figure 16). These findings could be a useful characteristic marker to differentiate xylazine from other illicit drugs.

The formation of the blue colour suggested that the reaction was not due to the presence of selenious acid having been reduced to elemental selenium, as no red or red‐brown precipitate formed during or after the primary colour formation [52]. A blue colour in the Mecke test, like that produced in our hands when testing xylazine, is known to be produced when pyrrole reacts with selenious acid [53]. Previous research has shown that the Mecke reagent can affect several other mechanisms, depending on the drug substrate [24, 50]. The root cause of the colour change triggered by the presence of xylazine remains a part of future work.

### 4.5 Xylazine Versus Its Synthetic Intermediates

**FIGURE 17:**
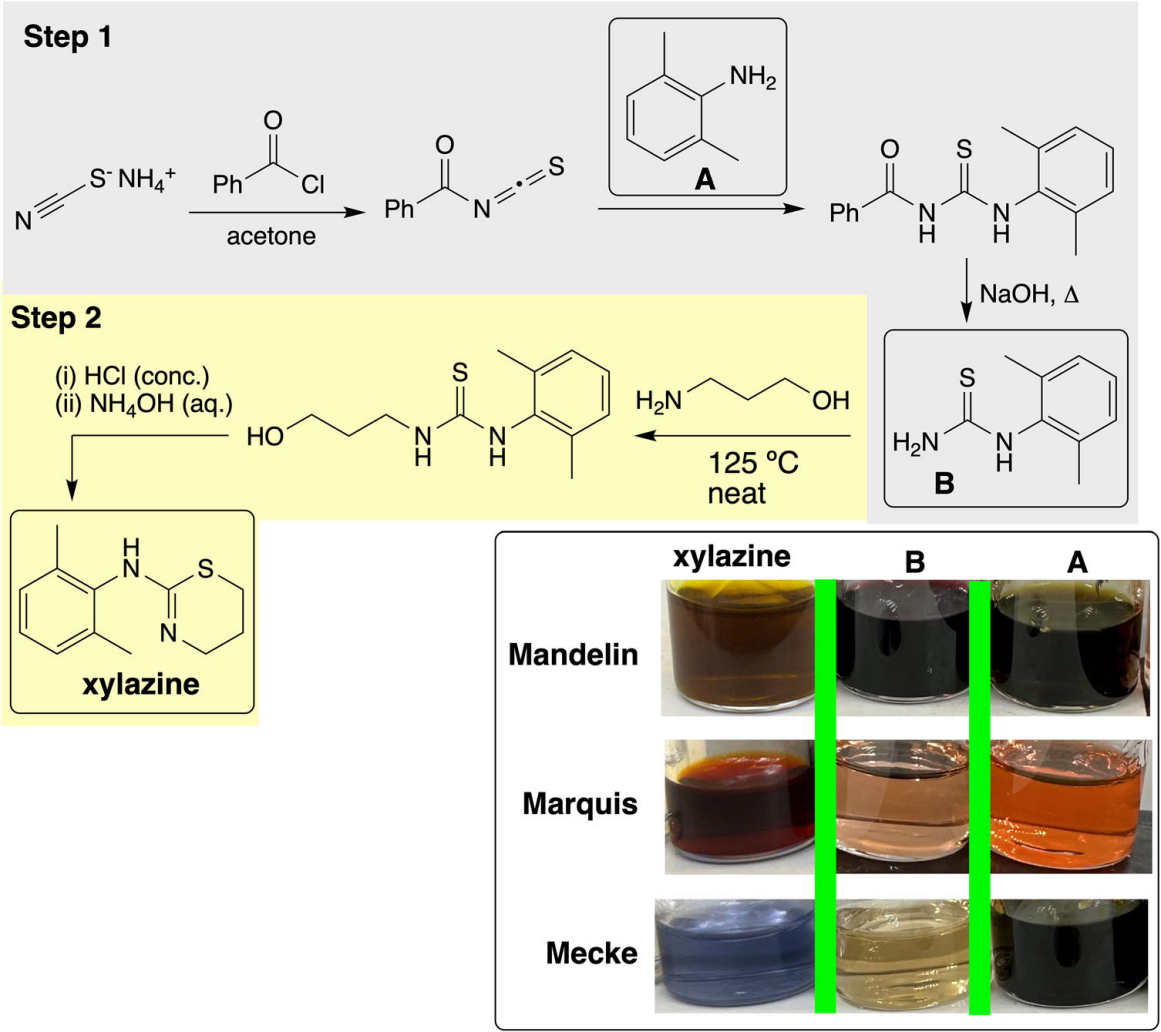
Synthesis of xylazine. The boxes highlight starting amine (A), the sole isolated intermediate (B), and xylazine in its free base form. Inset: Visual presumptive test results for aniline (A) and thiourea (B).

**FIGURE 18:**
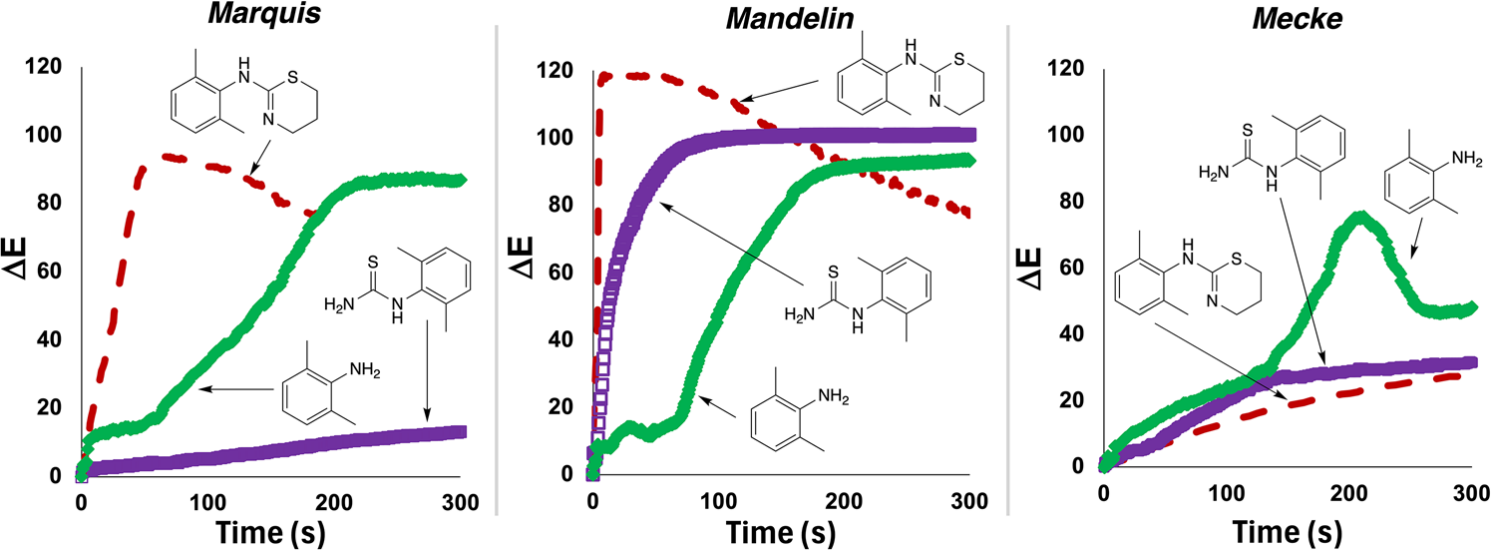
The differing time‐resolved behaviour of aniline (A) and thiourea (B) versus xylazine, using the Marquis, Mandelin and Mecke presumptive tests. All ∆*E* profiles were calculated by processing the reaction videos using *Kineticolor*.

Having established the separate and combined value of the Marquis, Mandelin and Mecke tests applied to xylazine, we investigated how the drug performed versus synthetic intermediates involved in its synthesis. To do this, we made some xylazine in‐house, following a patent‐published route (Figure 17) [46]. Thus, we were able to compare the by‐eye and *Kineticolor* test results of xylazine versus both the intermediate thiourea (B) and the parent aniline (A) used to make that intermediate. For all three presumptive test formulations, aniline A and thiourea B gave different colorimetric results versus xylazine (Figure 17). The time‐resolved behaviour of the aniline and thiourea in question under the spot test conditions already applied to xylazine further emphasised the distinctiveness of the drug versus the compounds used to make it (Figure 18).

### 4.6 Testing Street Samples and Drug Cocktails

**FIGURE 19:**
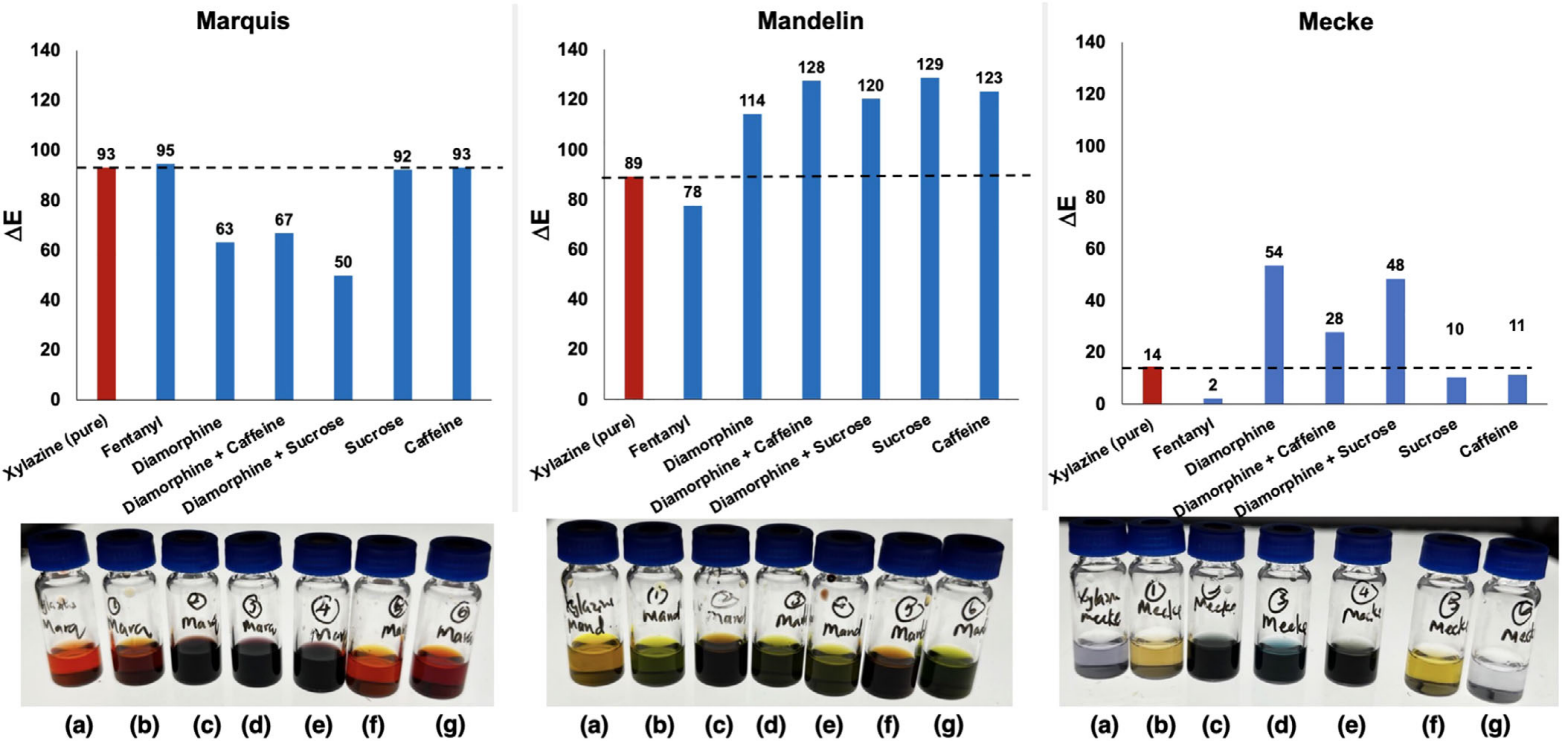
Top: ∆*E* response of various xylazine‐containing drug cocktails with the Marquis, Mandelin, and Mecke reagents. Bottom: Visual result of each test: (a) pure xylazine, (b) 2.5:1 with fentanyl, (c) 1:1 with diamorphine (heroin), (d) 1:1:1 with diamorphine and caffeine, (e) 1:1:1 with diamorphine and sucrose, (f) 1:1 with sucrose, and (g) 1:1 with caffeine.

We next investigated the response of adulterated xylazine samples with the same three‐test strategy, with a focus on fentanyl/xylazine and heroin (diamorphine)/xylazine combinations. For all three presumptive test reagents, pure xylazine produced a distinct colorimetric response from any of the ’cocktails’ employed. From the ∆*E* responses alone, the Mandelin and Mecke reagents triggered a more distinct range of colour changes than the Marquis reagent (Figure 19).

### 4.7 Limitations

Obtaining pure samples from crime scenes or clandestine laboratories is often challenging in forensic science. The commercially purchased xylazine used in this study had a purity higher than 99%. Therefore, testing xylazine mixed with other adulterants with existing colour presumptive test reagents would provide valuable further insight into detecting the presence of xylazine. From a video recording perspective, lighting presented a limiting factor in this study as *Kineticolor* (and other computer vision analysis) was influenced by the amount of light shining on the sample vial. Such environmental factors highlight additional usefulness in obtaining time‐resolved kinetic profiles for presumptive tests. Having said all of this, the data generated from the time‐resolved colours extracted from the spot test video footage, using *Kineticolor*, is so rich (providing many colour channel components in several colour languages, all time‐resolved) that the majority of these data are hosted in a supplementary data repository for the reader's consumption. Lastly, while computer vision has enabled the extraction of valuable time series from video recordings of the tests, the underlying mechanism(s) of the reactions between xylazine and the test reagents remains unclear. Indeed, future work is required to compare the response of xylazine versus other drugs that contain the isothiourea functional group. Structural confirmatory tests and deeper mechanistic investigations remain part of ongoing work.

## 5 Conclusions

The combination of the Marquis, Mandelin and Mecke reagents provides a potential strategy in spot testing for the presence of xylazine. The colour changes triggered by these tests, when exposed to xylazine, were yellow, blue and red, respectively. Video recording of these same presumptive tests revealed an additional layer of characteristic and time‐resolved chemical information than merely running the tests for single‐point analysis by‐eye. The colour changes from all three xylazine tests were approximated by‐eye using RAL charts, then quantified using the computer vision software, *Kineticolor*. The combination of colour changes demonstrated the potential to develop a three‐test colorimetric assay to identify xylazine from other unknown samples. The numerical expression of colours, ∆*E* and the time‐resolved ∆*E* profile, highlighted the possibility of digitally‐enhancing colour interpretation, transforming the presumptive tests from a series of subjective by‐eye assessments to an objective analysis tool in identifying xylazine and other illicit drugs. While these current findings are most directly applicable to laboratory settings, future work will focus on understanding how to translate these methods to field‐based applications.

## Author Contributions

**Hui Yun Chang**: data curation, formal analysis, investigation, visualization, writing–original draft. **Kristin Donnachie**: investigation, methodology, visualization. **Timothy J. D. McCabe**: methodology, supervision, validation, writing–review and editing. **Henry Barrington**: data curation, methodology, software, writing–review and editing. **Felicity Carlysle–Davies**: conceptualization, resources, supervision. **Kristin Ceniccola–Campos**: conceptualization, supervision, writing–review and editing. **Marc Reid**: conceptualization, data curation, formal analysis, funding acquisition, methodology, project administration, resources, software, supervision, validation, visualization, writing–original draft, writing–review and editing.

## Conflicts of Interest

MR is the inventor of *Kineticolor* and leading the software commercialization process. For information on licensing *Kineticolor* software, please contact the corresponding author and the University of Strathclyde technology transfer office.

## Data Availability

Machine‐readable files related to 3D printing CAD files, computer vision data, liquid handling robot protocols, and NMR analysis can be downloaded from figshare at: https://doi.org/10.6084/m9.figshare.26564395.v2.
